# Health Administration by County Councils

**Published:** 1921-08-13

**Authors:** 


### Health Administration by County Councils.

Sundry points of importance regarding health and administration were discussed at the quarterly meeting of the County Councils' Association.

At the request of the Association of Inspectors of Midwives, it was decided to request the Health Ministry to take steps to institute the registration and inspection of lying-in homes by the County Councils and County Borough Councils, subject to the proviso that delegation to other authorities of the duty sought to be so conferred be not permitted.

### Medical Practitioners and Midwifery Fees.

Regarding the extent of the liability of local supervising authorities in regard to the payments to medical practitioners called in cases of emergency under the Midwives Act, 1918, the Association came to the conclusion that the proposal understood to have been made by the Health Ministry (that the liability for payment should be limited to services performed by doctors within one month from the day of birth) affords insufficient protection to the authorities, who should, in cases where their medical officer has certified that the emergency contemplated by the Act had ended, be entitled to terminate their liabilty by notice to the medical practitioner concerned. The Dorset County Council drew attention to the difficulties involved by the absence of machinery for a systematic inquiry into the financial circumstances of patients or for the collection of fees from them; but the Association took the view that it is inadvisable, in view of the variation of local conditions, to attempt to establish any uniform machinery in regard to the collection of fees, and that each local authority should therefore make such arrangements in the matter as may be best suited to its individual requirements.

### Extra Nourishment for Tubercular Patients.

West Riding County Council submitted a protest against the attitude of the Health Ministry in limiting the amount to be spent on extra nourishment for tuberculous persons to £2 per 1,000 of the population; but the Association was not prepared, as a matter of general principle, to disagree with the limit, though at the same time it considered that special consideration should be given to applications for relaxation of the general rule in cases where the local authority could prove that £2 per 1,000 was inadequate.

